# Effect of various decontamination procedures on disposable N95 mask integrity and SARS-CoV-2 infectivity

**DOI:** 10.1017/cts.2020.494

**Published:** 2020-06-11

**Authors:** Jeffrey S. Smith, Haley Hanseler, John Welle, Rogan Rattray, Mary Campbell, Tacy Brotherton, Tarsem Moudgil, Thomas F. Pack, Keith Wegmann, Shawn Jensen, Justin Jin, Carlo B. Bifulco, Scott A. Prahl, Bernard A. Fox, Nicholas L. Stucky

**Affiliations:** 1 Providence Portland Medical Center, Department of Medicine, Portland, Oregon, USA; 2 OHSU Medical School, Portland, Oregon, USA; 3 Regional Pathology, Providence St. Joseph Health, Portland, Oregon, USA; 4 Cancer Research Animal Division, Robert W. Franz Cancer Center, Earle A. Chiles Research Institute, Providence Cancer Institute, Portland, Oregon, USA; 5 Molecular & Tumor Immunology, Robert W. Franz Cancer Center, Earle A. Chiles Research Institute, Providence Cancer Institute, Portland, Oregon, USA; 6 Axovant Gene Therapies, Ltd, Durham, North Carolina, USA; 7 Translational Molecular Pathology and Molecular Genomics, Robert W. Franz Cancer Center, Earle A. Chiles Research Institute, Providence Cancer Institute, Portland, Oregon, USA; 8 Electrical Engineering and Renewable Energy, Oregon Institute of Technology, Wilsonville, Oregon, USA; 9 Department of Molecular Microbiology and Immunology, and Knight Cancer Institute, Oregon Health and Science University, Portland, Oregon, USA

**Keywords:** Infectious diseases, mask decontamination, SARS-CoV-2, COVID-19, vaporized hydrogen peroxide, ultraviolet germicidal irradiation, N95

## Abstract

**Introduction:**

The COVID-19 pandemic has created a high demand on personal protective equipment, including disposable N95 masks. Given the need for mask reuse, we tested the feasibility of vaporized hydrogen peroxide (VHP), ultraviolet light (UV), and ethanol decontamination strategies on N95 mask integrity and the ability to remove the infectious potential of SARS-CoV-2.

**Methods:**

Disposable N95 masks, including medical grade (1860, 1870+) and industrial grade (8511) masks, were treated by VHP, UV, and ethanol decontamination. Mask degradation was tested using a quantitative respirator fit testing. Pooled clinical samples of SARS-CoV-2 were applied to mask samples, treated, and then either sent immediately for real-time reverse transcriptase–polymerase chain reaction (RT-PCR) or incubated with Vero E6 cells to assess for virucidal effect.

**Results:**

Both ethanol and UV decontamination showed functional degradation to different degrees while VHP treatment showed no significant change after two treatments. We also report a single SARS-CoV-2 virucidal experiment using Vero E6 cell infection in which only ethanol treatment eliminated detectable SARS-CoV-2 RNA.

**Conclusions:**

We hope our data will guide further research for evidenced-based decisions for disposable N95 mask reuse and help protect caregivers from SARS-CoV-2 and other pathogens.

## Introduction

A novel human coronavirus that is now named severe acute respiratory syndrome coronavirus 2 (SARS-CoV-2) emerged from Wuhan, China, in December 2019^1^ and quickly resulted in a global pandemic. The rapid spread of SARS-CoV-2 has created a high demand on personal protective equipment (PPE), and many hospitals worldwide are facing severe shortages. As transmission of SARS-CoV-2 occurs primarily through respiratory droplets, procedure masks and disposable N95 masks in particular have faced severe supply shortages. Contrary to manufacturer recommendations, this unprecedented pandemic has required reuse of these masks. Indeed, many frontline healthcare workers have adopted individualized mask decontamination strategies with unclear effects on mask integrity and on SARS-CoV-2 decontamination efficacy. Due to more limited supply, more stringent production requirements and requirement for critical lifesaving aerosol generating procedures N95 masks have become a priority in our health system. This team was tasked with determining feasibility of mask decontamination. Prior studies have investigated how decontamination procedures, including ethanol, ultraviolet light (UV), and vaporized hydrogen peroxide (VHP) alter N95 mask integrity,^2–8^ but it is unclear how effective these sterilization procedures are at destroying SARS-CoV-2. Here we investigate the effect of different decontamination methods on disposable N95 mask integrity and on eliminating the infectious potential of SARS-CoV-2.

## Materials and Methods

Briefly, disposable N95 masks, including medical grade (1860, 1870+) and industrial grade (8511) masks, were tested for mask integrity using a quantitative respirator fit testing with a Portacount Pro 8030. The masks were treated by three methods (70% ethanol, UV, and VHP), and mask degradation was measured after treatment. Pooled clinical samples of SARS-CoV-2 were applied to mask samples and treated by the above methods. After treatment, the samples were each immersed in cell culture media which was either sent immediately for real-time reverse transcriptase–polymerase chain reaction (RT-PCR) or incubated with Vero E6 cells for 4 days and then sent for RT-PCR to assess SARS-CoV-2 viability. Detailed methods are available in supplemental materials.

## Results

**Fig. 1.**
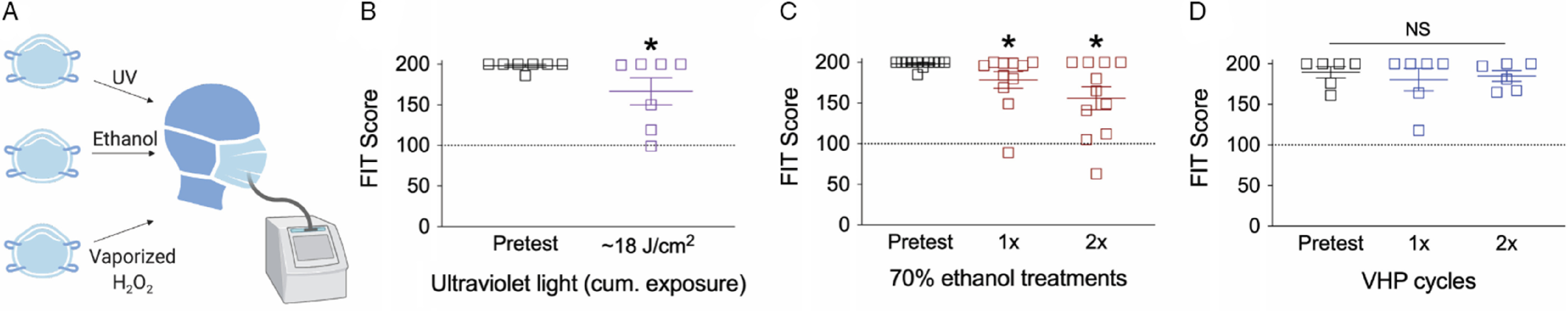
Effect of decontamination methods on N95 mask integrity. (A) Cartoon of N95 mask decontamination methods. Effect of (B) UV light, (C) two applications of 70% ethanol, or (D) two treatments of VHP on disposable N95 mask integrity. For panel B, *P < 0.05, one-tailed t-test; for panels C and D, *P < 0.05, one-way ANOVA with Fischer LSD post hoc one-tailed analysis relative to pretest condition. Dashed line at 100 indicates an acceptable FIT score. NS, not significant.

We first investigated if decontamination strategies, such as 70% ethanol, UV, or VHP, affected N95 mask integrity. We assessed N95 mask integrity through quantitative respirator fit testing (Fig. 1A). Quantitative fit testing measures particle concentration inside and outside the respirator and calculates a “FIT score,” the ratio of the two measurements. A FIT score of ≥100 is considered sufficient protection from aerosolized particles. Both repeated 70% ethanol exposure and extended exposure to UV significantly impaired mask integrity as assessed by FIT scores, consistent with prior reports, although the average score remained above an acceptable functional threshold of 100 in both conditions (Fig. 1B,C). VHP maintained an average FIT score of ≥100 with minimal, nonstatistically significant degradation of mask components (Fig. 1D). A single treatment of 70% ethanol noticeably impaired mask function, even when masks felt dry to the touch (Fig. 1C). N95 mask integrity was more greatly impaired at 30 min than 4 h (Supplemental Fig. 1). Results were consistent across N95 mask subtypes for both repeated ethanol exposure (Supplemental Fig. 2), high-intensity ethanol exposure (Supplemental Fig. 3), and VHP (Supplemental Fig. 4).

**Fig. 2.**
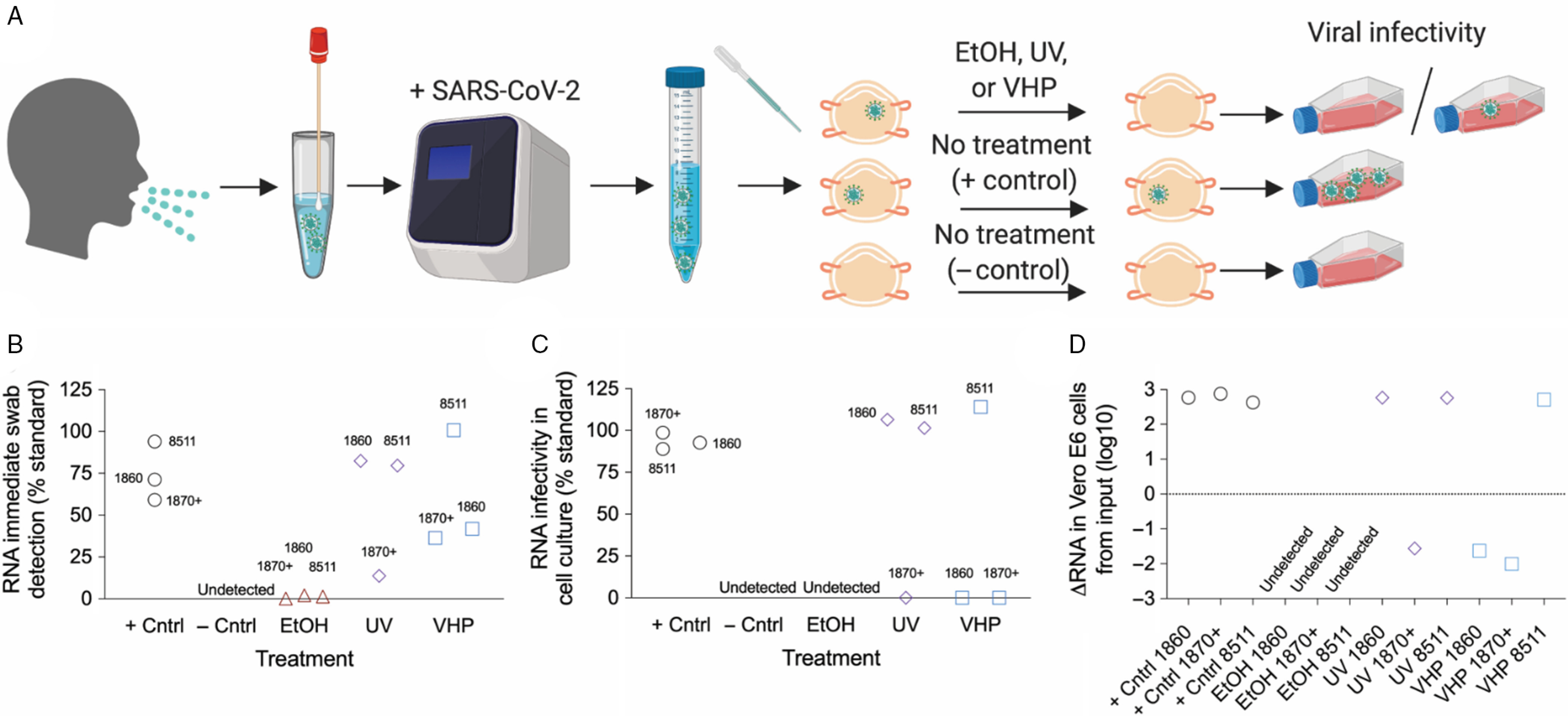
Effect of decontamination methods on SARS-CoV-2 infectivity. (A) Cartoon of SARS-CoV-2 decontamination experimental design. (B) RNA detected on the surface of N95 masks immediately after the indicated decontamination treatment. (C) Infectivity of SARS-CoV-2 in Vero E6 cells after masks underwent the indicated decontamination treatment. (D) Relative Log10 change of RNA isolated from immediate detection and then detected after infectivity of SARS-CoV-2 culture as assessed by cycle threshold. RNA data displayed is from the SARS-CoV-2 envelope primer set. Data from other primer sets, as well as cycle threshold data, are available in Table 1. For B, Data are normalized for the starting SARS-CoV-2 inoculum; for C, data are normalized to the inoculum directly placed in Vero E6 cell culture. Results are from a single experiment.

We next tested if decontamination with 70% ethanol, UV, or VHP changed viral RNA levels or viral infectivity. In this single experiment, six patients within our hospital system with the highest SARS-CoV-2 titer obtained by nasopharyngeal swab (as assessed by quantitative polymerase chain reaction [qPCR] cycle threshold) were pooled and applied to portions of 1860, 1870+, or 8511 disposable N95 masks and straps, except in the case of the negative control where no virus was applied (Fig. 2 A). N95 masks subsequently underwent decontamination, aside from the positive controls that were set aside. Following decontamination, N95 masks were immersed in ~3 mL of cell culture media. The media was then sterile filtered, and residual SARS-CoV-2 RNA assessed by RT-qPCR with five different primer sets selective for SARS-CoV-2. SARS-CoV-2 RNA was detected on all masks exposed to virus (Fig. 2B, Table 1). However, as the presence of viral RNA does not necessarily indicate viable virus, we then tested the infectious potential of any remaining viable virus exposed to each decontamination condition by applying a fraction of remaining media to Vero E6 cells. We proceeded to culture the virus with cells for 4 days before extracting media to test for the presence of SARS-CoV-2 by both RT-qPCR (Fig. 2C, Table 1) and semi-quantitative viral-induced cytopathic effects (Supplemental Fig. 5).

**Table 1.** Cycle threshold for values of five primer sets for each experimental condition. RNase P is used as an indicator in clinical specimens that sufficient human cellular material was collected, as well as an extraction/procedural control, and was included as reference. RNase P was likely present on N95 masks from skin contact during handling

Sample	E-gene (Ct)	N-gene (Ct)	CDC N1 (Ct)	CDC N2 (Ct)	CDC N3 (Ct)	RNase P (Extraction Control) (Ct)
**SARS-CoV-2 initial RNA**						
REF	20.67	26.67	19.1	18.45	19.33	32.1
ETOH 1860	26.23	32.63	25	28.1	26.1	33.2
ETOH 1870+	30.33	37.01	28.4	33.1	30.4	33.5
ETOH 8511	26.9	32.62	25.2	28.5	26.5	33.3
+ Cntrl 1860	21.16	27.28	19.5	19.9	19.7	31.3
+ Cntrl1870+	21.43	27.19	19.4	19.9	19.6	30.9
+ Cntrl 8511	20.76	26.72	19	19.4	19.4	32.5
UV 1860	20.95	27.14	19.2	19.9	19.8	33.2
UV Cntrl 1870+	23.54	29.78	21.2	22.1	21.6	34.7
UV Cntrl 8511	21	27.3	19.3	19.9	19.5	34.3
VHP 1860	21.93	28.57	20.2	20.6	20.6	32.8
VHP 1870+	22.13	28.68	20.4	21	20.8	31.6
VHP 8511	20.66	27.01	19	19.5	19.3	31.5
- Cntrl	Undetected	Undetected	Undetected	Undetected	Undetected	36.1
**SARS-CoV-2 cell culture infectivity RNA**						
REF	11.85	15.96	10.7	11	11.1	32.9
ETOH 1860	Undetected	Undetected	Undetected	Undetected	Undetected	43.3
ETOH 1870+	Undetected	Undetected	Undetected	Undetected	Undetected	41.7
ETOH 8511	Undetected	Undetected	Undetected	Undetected	Undetected	40.1
+ Cntrl 1860	11.96	15.72	10.8	11.1	11.2	34.4
+ Cntrl 1870	11.87	15.8	10.7	11.1	11.2	34.3
+ Cntrl 8511	12.02	16.32	10.9	11.2	11.3	35
UV 1860	11.76	15.83	10.6	11	11.1	35.7
UV Cntrl 1870+	28.73	34.04	25.1	26.6	26.8	38.6
UV Cntrl 8511	11.83	15.88	10.7	11.1	11	33.6
VHP 1860	27.33	33.22	24.6	26.2	26.1	39
VHP 1870+	28.79	33.23	25.3	25.9	25.9	38.3
VHP 8511	11.66	15.75	10.7	11.1	11.1	33.6
- Cntrl	Undetected	Undetected	36.5	35.1	Undetected	34.5

In descriptive analyses, all three N95 mask types in the positive control cell culture infectivity study had substantially lower cycle thresholds (higher amount of virus) than RNA detected immediately after decontamination corresponding to approximately three log-fold increase in SARS-CoV-2 RNA. N95 masks undergoing different decontamination strategies showed variation in RNA levels (Fig. 2D). No RNA was detected in cell culture in any of the three masks treated with 70% ethanol. Supporting the selectivity of our primers, no SARS-CoV-2 RNA was detected in any negative control sample in the initial media by any of the five primer sets. The two most sensitive primers did detect low amounts of viral RNA (Ct >35) in the infectivity negative control, likely as a result of slight contamination.

## Discussion

In this study, we investigated the effect of different decontamination methods on disposable N95 masks for virucidal effect on SARS-CoV-2 and on N95 mask integrity. This study was initiated early in the course of COVID-19 outbreak when supplies of disposable N95 masks at our institution were limited. Without knowing whether it would be possible to procure new masks, healthcare providers like ourselves had to make urgent decisions about how best to decontaminate existing N95 masks with limited data on decontamination strategies targeting SARS-CoV-2. In order to be compatible with reuse, methods of N95 mask SARS-CoV-2 decontamination must remove the viral threat, be harmless to the mask user, and not compromise the integrity of the various mask elements. The decontamination methods utilized, 70% ethanol, UV, and VHP have previously been demonstrated to be safe for mask users.^3–5,9^ We found that any ethanol exposure significantly altered mask integrity, as previously reported.^7^ We also found that the impact of 70% ethanol on mask integrity appears time dependent. In fact, 30 min after 70% ethanol application there was even a larger decline in measured integrity, even though the N95 masks felt dry to the touch. Consistent with prior studies, we did observe a decline in SARS-CoV-2 infectivity (as assessed by Vero E6 culture) after certain decontamination strategies.

Depending on perspective, the high concentration of SARS-CoV-2 initially applied to N95 masks can be considered either a strength or a weakness. Without access to sophisticated droplet or aerosol generating machines and to avoid unnecessary risk, SARS-CoV-2 containing media was applied directly to the mask samples with a pipette. We intended to deliver the highest challenge possible to assess decontamination efficacy. Samples from the 6 highest titer patients in our healthcare system to date were pooled, and 100 uL of this concentrated SARS-CoV-2 containing media was directly infiltrated into the N95 masks with the attempt to expose the middle layer. It is hard to imagine a realistic scenario where healthcare workers would face this degree of mask inoculum. Methods able to decontaminate N95 masks under these intense exposure conditions would likely be highly efficacious in actual practice. However, methods that appear less effective in decontaminating SARS-CoV-2 in our experiment, such as UV, would almost certainly be more effective if masks were challenged in a more realistic exposure scenario. Another possible reason that UV treatment appeared virucidal in only one of the masks we tested is that we chose a dose in the lower of the range of those previously shown to be virucidal.^10^ In one study, a dose of 0.5 J/mc2 was less virucidal than a dose of 1 J/cm^2^.^11^ Regarding VHP treatment, while all masks treated with VHP did not completely eliminate infectious SARS-CoV-2 RNA, the two healthcare grade masks did show an approximately five log10 reduction in SARS-CoV-2 RNA relative to the positive control. Further work with additional time points is necessary to confirm if this RNA is infectious or not. In comparison, such a log10 reduction in infectivity would exceed the ‘99.97%’ germicidal efficacy quoted by some hand sanitizers and exceeds the three log10 reduction estimated to fully decontaminate a mask in an influenza model.^12^ As mentioned above, further work across multiple institutions would be necessary to confirm the degree of this germicidal effect. Overall, it is possible our data inadvertently underestimate the decontamination efficacy of some methods.

Ethanol treatment did lead to demonstrate sufficient virucidal activity but impaired mask integrity at the treatment dose tested, most notably 30 min after ethanol treatment. Nevertheless, 4 h after ethanol treatment also demonstrated impaired mask integrity, albeit less than at 30 min. It is possible that ethanol-induced disruption of electrostatic charge on N95 filters can be at least partially recovered, although further work is necessary to test this hypothesis. For ethanol treatment, it is possible that there might be an intermediate treatment dose which balances the virucidal activity with less, and possibly acceptable, impaired mask integrity. In fact, given the variable virucidal and degradation results evidenced by all three treatments, there may be a combinatorial decontamination strategy that could be pursued with success. To our knowledge, there is no such strategy currently being assessed.

## Limitations

There are many limitations to this study. First and foremost experiments to measure SARS-CoV-2 RNA and infectivity were conducted only once. Our project was initiated to inform decision makers about strategies for mask decontamination within a narrow timeframe. Due to limited resources including continued access to BSL3 laboratory space only a single SARS-CoV-2 decontamination experiment was performed. Another limitation is that clear variation exists between N95 mask type and decontamination efficacy. This could be due to technical replicate variation. However, a reasonable hypothesis to test is that N95 masks with a fluid-resistant coating (healthcare grade) relative to the 8511 (non-healthcare grade) results in less viral uptake and are therefore more likely to be effectively decontaminated. More work is needed to understand this relationship between N95 mask material and decontamination efficacy. Notably, we did not test if ethanol, UV, or VHP impaired N95 fluid-resistant coating on healthcare grade masks. Next, FIT testing, which is utilized by our healthcare system to determine N95 integrity, could in some instances underrepresent actual protection. Likewise, it is also possible ethanol or UV may have resulted in internal mask degradation, producing particulate that may have overestimated the negative impact on N95 mask integrity. While we believe our quantitative FIT testing provides a reasonable estimate of N95 mask function that can aid in comparing imperfect decontamination strategies, we did not conduct the plethora of gold-standard National Institute for Occupational Safety and Health assays required for validation. Another limitation is that we did not test how time alone impacts SARS-CoV-2 infectivity. It is entirely possible that no decontamination, and allowing for decay of virus infectivity over time, is a preferable strategy when faced with no ideal options.

Reuse of disposable N95 masks after decontamination has not been recommended under typical circumstances.^3^ New masks are always preferred. Nevertheless, we recognize that healthcare systems and frontline providers around the world are currently facing unprecedented shortages of protective respiratory devices and must make decisions that are not ideal. The USA Food and Drug Administration recently authorized emergency use of VHP as a mask decontamination method,^13^ and our results are consistent with others which showed no significant degradation of mask integrity after two cycles of VHP.^6,8^ We hope our data will help guide evidenced-based decisions and future experiments that will protect healthcare providers fighting SARS-CoV-2 and other pathogens.

## Supplementary material

For supplementary material accompanying this paper visit https://doi.org/10.1017/cts.2020.494. click here to view supplementary material
