# Supplementary material for: Effect of various decontamination procedures on disposable N95 mask integrity and SARS-CoV-2 infectivity
Source: J Clin Transl Sci. 2020 Jun 11;5(1):e10. doi: 10.1017/cts.2020.494 (PMC7605403; doi:10.1017/cts.2020.494)
Supplement: Supplementary file 1 [file S205986612000494Xsup001.docx]

**Supplemental Material**

**Detailed Methods**

**N95 masks**

Disposable N95 masks variants tested included 1860, Aura 1870+, and industrial 8511 were manufactured by 3M (St. Paul, Minnesota, USA). K-N95 disposable masks were manufactured by Jiande Chaomei Daily Chemicals (Mainland China). Masks produced by 3M were combined in main text analysis, with subgroup analyses provided within the supplemental figures. No K-N95 masks had a pretest FIT score of ≥100 in our hands, and therefore were not included in experiments summarized in the main text.

**Quantitative evaluation of N95 mask integrity**

Quantitative respirator fit testing of disposable N95 masks were conducted using a Portacount Pro 8030 (TSI, Minnesota). In brief, N95 masks were cannulated and an external, air-tight tube attached between the Portacount and mask. Particles that entered the Portacount pro passed through a saturator tube, combined with ethanol vapor, and passed through a condenser tube, effectively enlarging the surface area of the particles. Droplets then passed through a laser, scattering light then quantified by a photodetector. This method allows for detection of particles of various sizes and is not limited to larger particulate. The mask operator then completed up to seven different activities wearing the disposable N95 mask, including normal breathing, deep breathing, head side-to-side, head up-and-down, bending over, jogging, and speaking. Not significant pretest variability existed between users of 3M masks (Supplemental Figure 6). A relative ratio of ambient air particle counts to intramask particle counts was taken, with a ceiling ratio of 200 for each activity. Since few particles should penetrate the high efficiency filter of functional disposable N95 masks, any particles found inside the respirator were conservatively attributed to either face seal leakage or disruption of mask filtration components. An objective ‘fit score’ was then calculated by the formula FIT score = activities/[(1/FF_1)_+(1/FF_2_)…1/FF_7_). Scores of 100 or greater (two log10 reduction in particulate) is considered sufficient for provider projection. Assessments of masks were controlled both intramask and intraoperator, unless otherwise noted.

**70% Ethanol**

70% ethanol obtained by mixing ethanol with 30% deionized water. For disposable N95 mask integrity assessments, 70% ethanol was sprayed 10 times on the mask exterior, the mask was flipped, and sprayed an additional 5 times on the interior, similar to the Italian protocol (14). For overnight application, masks were saturated with 70% ethanol, placed in a sealed plastic bag overnight, removed in the morning and allowed to airdry for ~8 hours prior to FIT testing.

**Vaporized hydrogen peroxide (VHP)**

Masks were decontaminated with 30% vaporized hydrogen peroxide using a protocol similar to that previously described (6). Briefly, disposable N95 masks were placed on a metal rack, exterior surface facing upwards. A Bioquell Z vaporizer (Andover, United Kingdom) utilizing 30% hydrogen peroxide solution (Sigma Aldrich, St. Louis, USA) was programmed to gas for 20 minutes at 10 grams per minute, reaching ~500 peak parts per million. Dwell at 4 grams per minute ran for 60 minutes maintain ~420 ppm throughout the full 60 mins. Aeration ran for 210 minutes until reaching safe entrance levels of 1ppm or less. Ambient room temperatures ranged from approximately 22°C ambient to 26°C, and the vaporizer component maintained 120°C during both the gas and dwell phases. Relative room humidity ranged from 38% to 99.5%.

**Ultraviolet light**

N95 masks were placed in biosafety cabinet with exterior surface facing towards the UV-C (General Electric 30W Germicidal T8 bulb emitting primarily at 254nm) light source and then flipped to face the interior surface toward the UV source to treat both sides. UV power was measured with a PowerMax-USB PS19 Power Sensor (Coherent Inc, Santa Clara CA) with and without a Schott WG305 filter at the site of mask placement.  The difference in the measurements was the UV-C irradiance.  Total UV dose was calculated using sensor surface area and time by the equation: irradiance x time = UV dose. To assess mask integrity, a UV dose of 18.4 J/cm2 (16 hrs) delivered to the exterior surface and 4.6 J/cm2 (4 hrs) delivered to interior surface was used to approximate multiple treatments. To assess virucidal efficacy each side of the mask samples were treated with a UV dose of 0.63 J/cm2 (33 min). This dose was chosen to be in the middle of dosing ranges presented in prior literature of 0.005-1 J/cm2.There was a significant range in prior literature which shows doses of 0.005 J/cm2 can inactivate coronaviruses on surfaces (15) but doses of 0.5-1 J/cm2 are used for decontamination of influenza (16). We chose a dose which we anticipated would balance efficacy and time which is consistent with other protocols currently in use (Lowe, John J., et al. "N95 filtering facepiece respirator ultraviolet germicidal irradiation (UVGI) process for decontamination and reuse." Nebraska Medicine March 21 (2020).).

**Vero cells and cell culture**

Vero cells were obtained from Drs. Victor DeFilippis and Hans-Peter Raue, Vaccine and Gene Therapy Institute, Oregon National Primate Research Center, OHSU. Vero cells were cultured in DMEM 1X, CAT# 1960-044, Lot# 2120580 (Gibco) with 4500 mg/L D-glucose (Gibco) with 10% FBS Cat# PS-500A, Lot# 31C141 (PEAK Serum) 2mM L-glutamine (Lonza) and 50 ug/ml gentamicin sulfate (Lonza). Cells were incubated at 37^o^C in a humidified incubator with 5% CO_2_. Cells were split 2 to 4 days prior to use and 10^5^ cells were plated into a T25 flask. All work with virus was performed in a certified biosafety cabinet in a BSL2+ negative pressure suite within the Earle A. Chiles Research Institute, Providence Cancer institute or the BSL3 suite of the Regional Pathology Laboratory, Providence St. Joseph Health, Portland, Oregon.

**SARS-CoV-2 detection**

After decontamination, N95 masks were immersed in ~3 mL of cell culture media gently agitated for 5 minutes. The media was then sterile filtered, and 600uL was transferred to flasks of Vero E6 cells for infectivity. The remaining approximately 400uL of sample was used for an estimate of SARS-CoV-2 RNA remaining after decontamination. Specific real-time reverse transcriptase–polymerase chain reaction (RT-PCR) targeting the nucleocapsid protein, envelope protein, and RNA-dependent RNA polymerase was used to detect the presence of SARS-CoV-2 similar to that previously described (17). 5 primer sets were used; primers previouslyvalidated to target the SARS-CoV-2 envelope and RNA-dependent RNA polymerase (18), as well as the threeCenters for Disease Control primers (N1, N2, N3) targeting the SARS-CoV-2 nucleocapsid protein. Nucleic acid extraction was performed using QIAsymphony DSP Virus/Pathogen Mini Kits (Qiagen). qPCR was performed and analyzed on a Roche cobas z480 analyzer or Applied Biosystems 7500 Fast Real-Time PCR Instrument. Equine Arteritis Virus (EAV) and RNase P (shown in table 1) were utilized as an RNA extraction control.

**SARS-CoV-2 viability**

Virus viability was assessed by end-point titration in Vero E6 cells similar to that previously described (15). Briefly, a nasopharyngeal swab was collected and placed in viral culture media. Positive SARS-CoV-2 samples were identified as above, and remaining SARS-CoV-2 positive samples with a low cycle threshold (high viral titer) were unthawed and pooled. SARS-CoV-2 positive viral media was transferred on dry ice to appropriate BSL conditions, dethawed, and combined with a saline/albumin mixture that roughly approximated the protein composition of human saliva. 100uL of this SARS-CoV-2 saliva-like solution was then applied to ~1” x 0.5” strips of 1860, 1870+, or 8511 disposable N95 masks, with filter components held together by a single staple. Gentle pressure was applied to increase SARS-CoV-2 saliva-like solution absorbance, and the sample was allowed to rest for 5 minutes. The positive control was transferred to a standard 50mL sterile falcon conical and submerged in DMEM culture media. Negative controls (N95 mask strips without application of SARS-CoV-2 saliva-like solution) were treated similarly. Samples undergoing sterilization were treated similarly as above; pipette application to cover entirety of both surfaces with 70% ethanol, ultraviolet light for 30 minutes per side, VHP, or set aside for 4hrs (positive control). After treatment samples were transferred to conical tubes with ~3 mL of cell culture media and were gently agitated intermittently for 5 minutes. Samples to be added to Vero E6 cells were then subsequently transferred via a 0.2 micron sterile filtration to test for infectivity. 600 µL of sample fluid was transferred to a total of 7 mL of media so all samples were diluted approximately 1:12.

**Specimens**

The Regional Providence Institutional Review Board approved the request to obtain leftover viral transport media from nasopharyngeal swabs found to be positive for SARS-CoV-2.

**
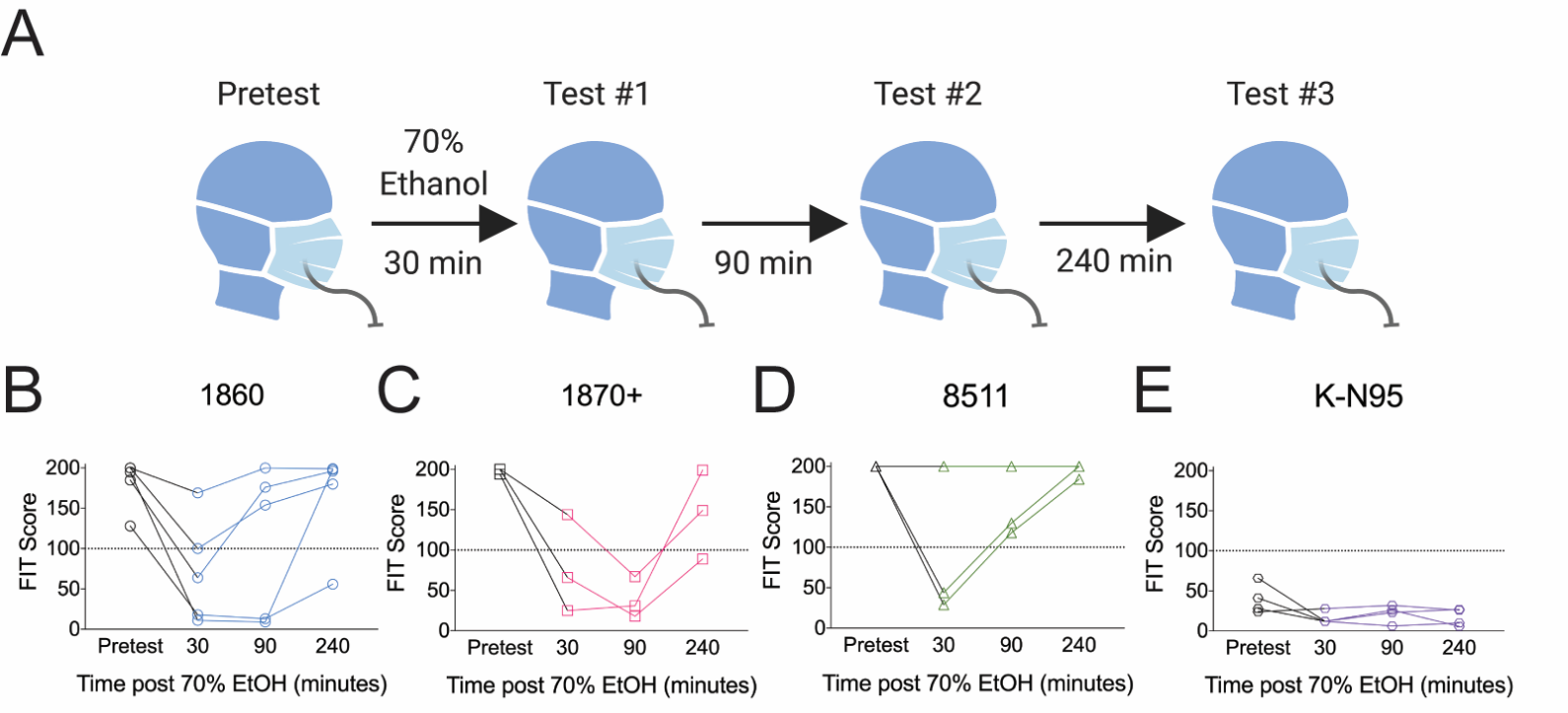
**

**Supplemental Figure 1: Effect of 70% ethanol treatment on N95 mask integrity is time-dependent**

**A)** Cartoon of ethanol treatment time course analyses. **B)** 1860, **C)** 1870+, **D)** 8511, or **E)** K-N95 masks were pretested, treated with 70% ethanol, and re-tested at the indicated time points. Individual N95 masks are shown. No K-N95 masks passed our FIT Pretest and were therefore not included in analyses shown in the main text.


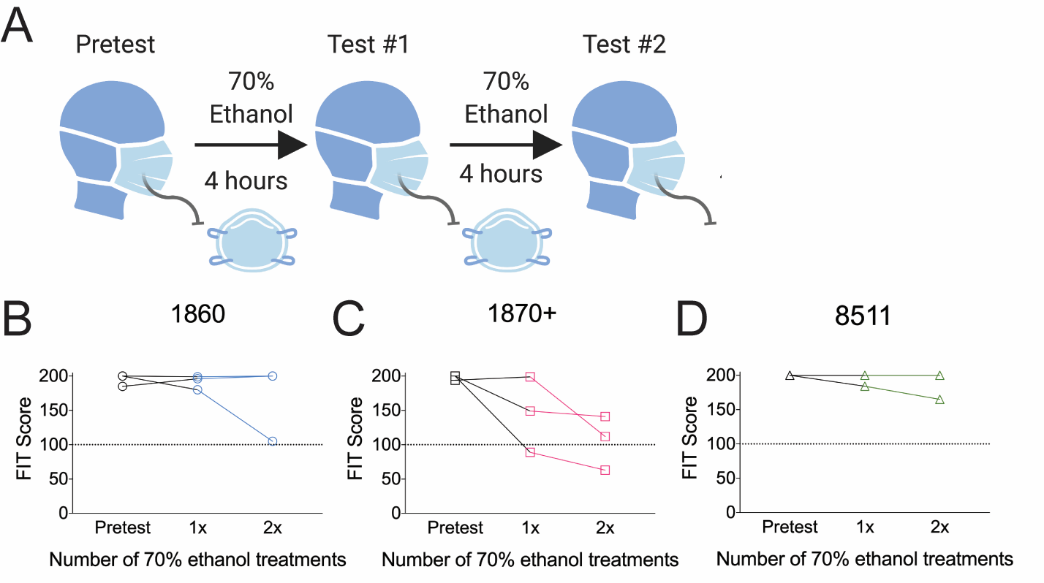


**Supplemental Figure 2: Effect of repeated ethanol on N95 mask integrity**

**A)** Cartoon of ethanol treatment time course analyses. **B)** 1860, **C)** 1870+, or **D)** 8511 were pretested, treated the indicated number of times with 70% ethanol, and re-tested. Individual N95 masks are plotted.


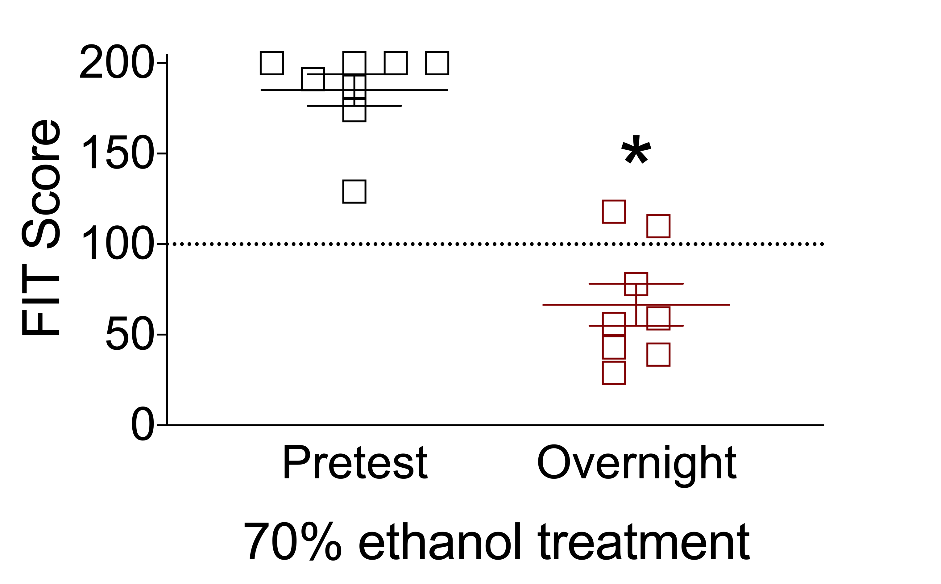


**Supplemental Figure 3: Overnight ethanol treatment impairs N95 mask integrity**

After completing FIT pretests, two 1860, three 1870+, and three 8511 disposable N95 masks were saturated with 70% ethanol, sealed within a plastic bag overnight, and allowed to air dry for 8 hours. Masks were then re-tested. Nose guards on some of the 1870+ masks were noted to have detached. *P<0.05, one-tailed t-test. Dashed line at 100 indicates an acceptable FIT score.

**
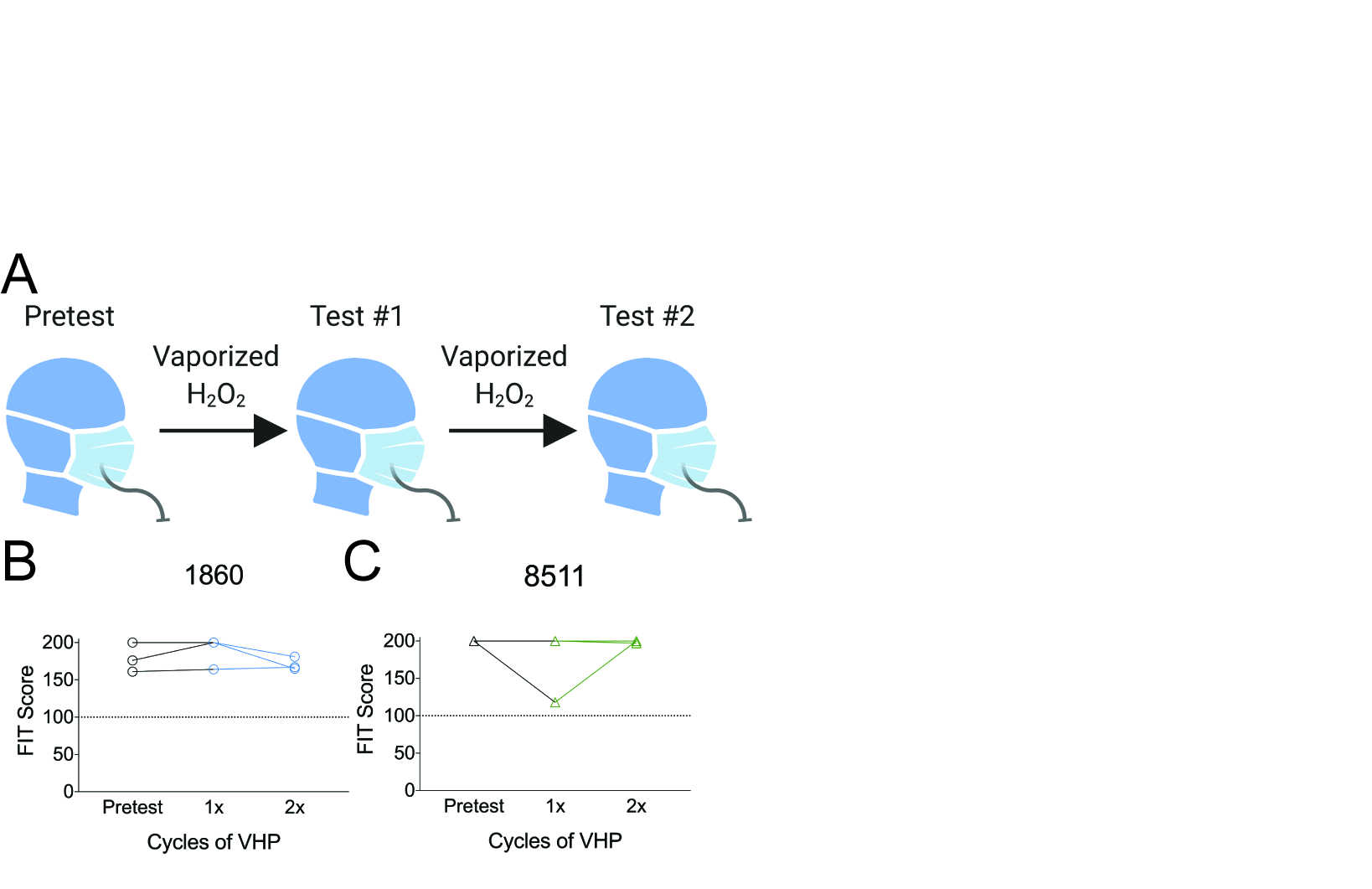
**

**Supplemental Figure 4: Effect of VHP on N95 mask integrity**

**A)** Cartoon of VHP treatment time course analyses. **B)** 1860 or **C)** 8511 N95 masks were pretested, treated the indicated number of cycles of 30% VHP, and re-tested. Individual N95 masks are plotted.

**
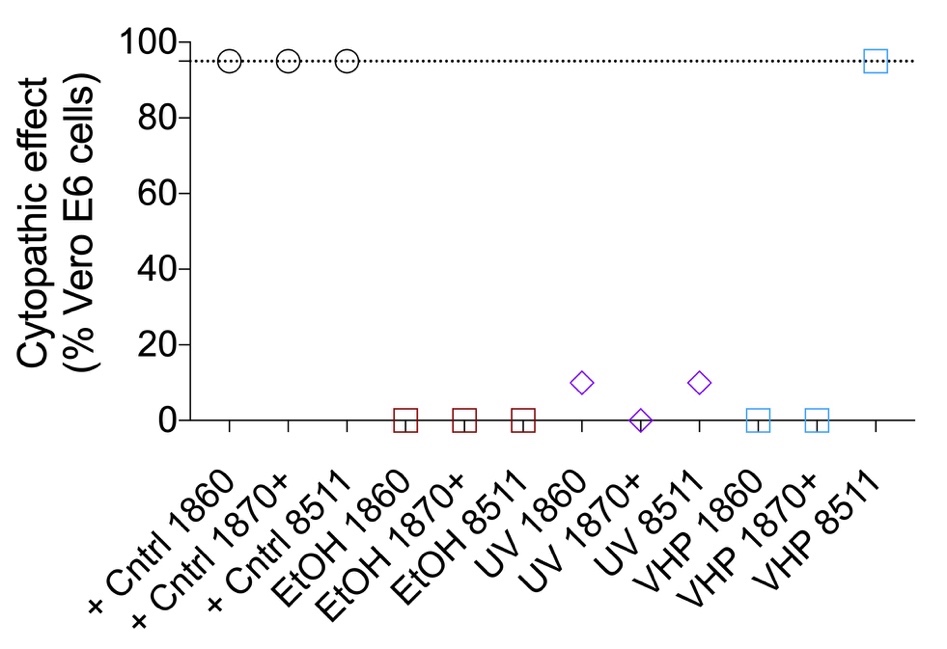
**

**Supplemental Figure 5**: **Cytopathic effect (CPE) of SARS-CoV-2 contaminated N95 masks on Vero E6 cells.** Vero E6 cells were analyzed under a standard microscope. Cell death in each condition was estimated based on visual inspection and recorded. Dash line represents the CPE of inoculum directly placed in Vero E6 cell culture (positive control).


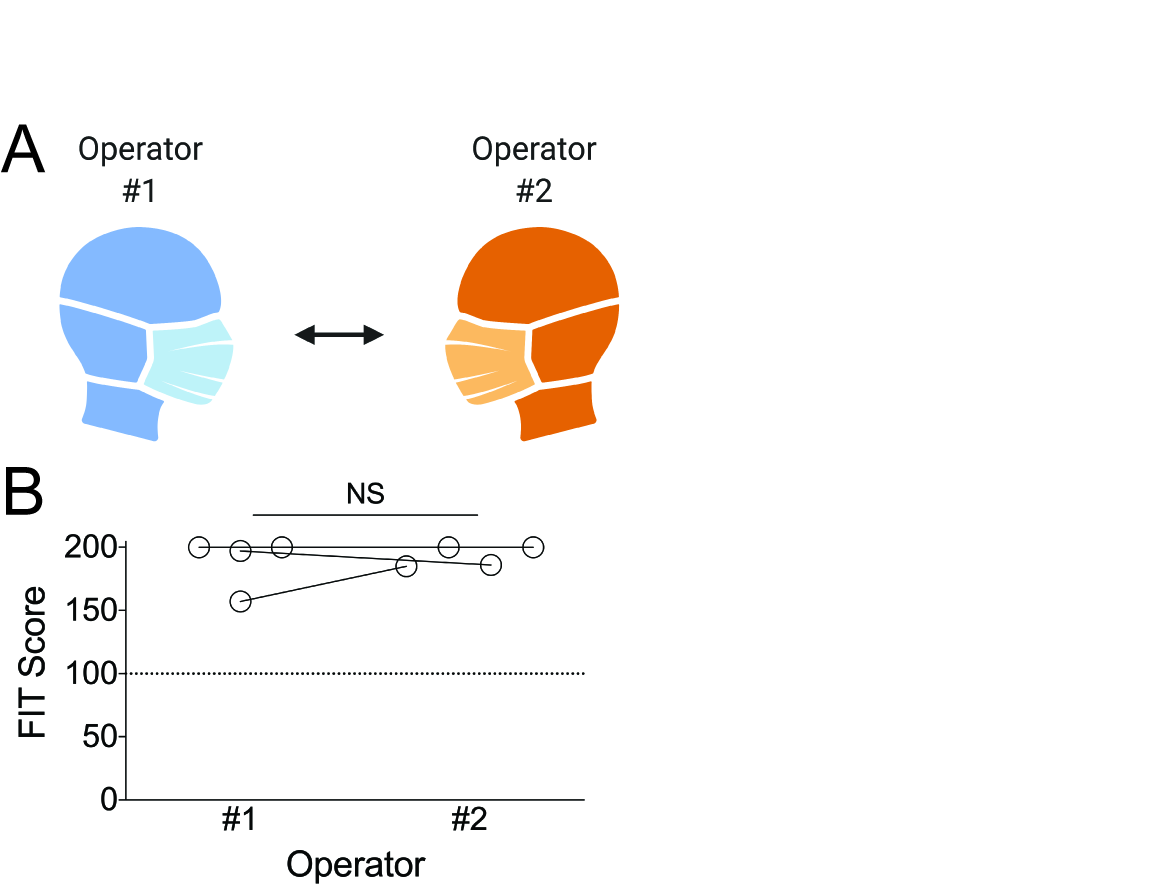


**Supplemental Figure 6: FIT scores are consistent between operators**

Two 1860 and two 1870+ masks were analyzed by two different operators. No significant difference in pretest FIT scores were observed. NS, not significant by student’s t-test.
